# Incidence of X and Y Chromosomal Aneuploidy in a Large Child Bearing Population

**DOI:** 10.1371/journal.pone.0161045

**Published:** 2016-08-11

**Authors:** Carole Samango-Sprouse, Eser Kırkızlar, Megan P. Hall, Patrick Lawson, Zachary Demko, Susan M. Zneimer, Kirsten J. Curnow, Susan Gross, Andrea Gropman

**Affiliations:** 1 Department of Pediatrics, George Washington University School of Medicine and Health Sciences, Washington, D.C., United States of America; 2 Natera Inc., San Carlos, California, United States of America; 3 Department of Neurology and Pediatrics, Children’s National Medical Center, Washington, D.C., United States of America; 4 The Focus Foundation, Davidsonville, Maryland, United States of America; University of Bonn, Institute of Experimental Hematology and Transfusion Medicine, GERMANY

## Abstract

**Background:**

X&Y chromosomal aneuploidies are among the most common human whole-chromosomal copy number changes, but the population-based incidence and prevalence in the child-bearing population is unclear.

**Methods:**

This retrospective analysis of prospectively collected data leveraged a routine non-invasive prenatal test (NIPT) using parental genotyping to estimate the population-based incidence of X&Y chromosome variations in this population referred for NIPT (generally due to advanced maternal age).

**Results:**

From 141,916 women and 29,336 men, 119 X&Y chromosomal abnormalities (prevalence: 1 in 1,439) were identified. Maternal findings include: 43 cases of 45,X (40 mosaic); 30 cases of 47,XXX (12 mosaic); 3 cases of 46,XX uniparental disomy; 2 cases of 46,XY/46,XX; 23 cases of mosaicism of unknown type; 2 cases of 47,XX,i(X)(q10). Paternal findings include: 2 cases of 47,XXY (1 mosaic); 10 cases of 47,XYY (1 mosaic); 4 partial Y deletions.

**Conclusions:**

Single chromosome aneuploidy was present in one of every 1,439 individuals considered in this study, showing 47,XXX; 47,XX,i(X)(q10); 47,XYY; 47,XXY, partial Y deletions, and a high level of mosaicism for 45,X. This expands significantly our understanding of X&Y chromosomal variations and fertility issues, and is critical for families and adults affected by these disorders. This current and extensive information on fertility will be beneficial for genetic counseling on prenatal diagnoses as well as for newly diagnosed postnatal cases.

## Introduction

X and Y chromosomal aneuploidies (the presence of an abnormal number of sex chromosome) are among the most common human whole-chromosomal copy number variations, with an estimated incidence in the general population between 1 in 400 to 1 in 1,000 [1,2–4] for each of the sex chromosome syndromes, with complex aneuploidies occurring far less frequently [5]. However, differences exist between newborn incidences and prenatal and adult incidences [6–8]. The population-based incidence and prevalence of the sex chromosomal aneuploidies (SCA) in the general adult population continue to be unclear. For example, prenatal incidence estimates vary for 47,XXY from between 1 in 500 [9] to 1 in 1,500 [2]. Women of advanced maternal age are more likely to receive prenatal testing, and since the probability of having an XXY child increases with maternal age, there may be an over representation of 47,XXY in prenatally diagnosed populations [2]. In postnatally diagnosed men, the prevalence of 47,XXY is reported to be 1 in 2,500 [2]. It is estimated that <10% of 47,XXY males are diagnosed before puberty, and <25% are ever diagnosed [10]. Additionally, only ~10% of 47,XXX girls are ever diagnosed [1]. Conversely, the majority of 45,X cases are diagnosed, though typically not until adulthood [11]. 45,X is a variety of monosomy, while the 47-chromosome karyotypes are trisomies. Monosomy and trisomy, respectively, refer to instances where 1 or 3 copies of a particular chromosome are present instead of the expected 2. Uniparental disomy (UPD) refers to when the expected 2 copies of a chromosome are present, but were both received from the same parent, rather than 1 from each.

Generally, SCAs are diagnosed postnatally, because of developmental or behavioral impairments or decreased fertility [12]. Prenatally, SCAs are usually diagnosed incidentally through invasive prenatal testing, such as amniocentesis or chorionic villus sampling, often performed in a high-risk pregnancy setting such as advanced maternal age, ultrasound anomalies, or a family history of a chromosomal disorder [13]. A new molecular genetic approach, that of non-invasive prenatal testing (NIPT), to detect fetal chromosomal anomalies in early pregnancy has provided another route to identify SCAs. NIPT works by analyzing cell-free fetal DNA (cffDNA) in maternal blood, and though the test is highly accurate, it is not diagnostic and requires cytogenetic confirmation. Mosaic SCAs, usually in the presence of a normal cell line, is also common. The most common sex chromosome mosaic cell lines include 45,X/46,XX, 46,XX/47,XXX, 46,XY/47,XXY and 46,XY/47,XYY. More complex SCAs, such as gain of more than 1 extra copy of the X or Y, and structural rearrangements of the X or Y occur far less frequently, whether seen as the sole cell line or in a mosaic form [5].

SCAs are derived from either de novo (new) mutations or structural rearrangements during meiosis of oogenesis or spermatogenesis, or during mitosis after fertilization [14]. Most autosomal aneuploidy is maternal in origin. However, approximately 80% of sex chromosome aneuploidies are paternal in origin: 6% of 47, XXX; 50% of 47, XXY; 80% of 45, X; and 100% of 47, XYY cases [15–17]. Studies have shown that an increased frequency of sperm aneuploidy is associated with male infertility [18].

As most SCA individuals are identified due to an associated finding, data on healthy SCA individuals in the general population is severely limited; therefore, population-based studies, which are independent of the varying rate of clinical ascertainment, are necessary to more accurately assess prevalence of SCA in the adult population and the impact on their fertility. Additionally, the fertility rates in the child-bearing adult population of individuals who themselves carry a sex chromosome abnormality has not been well defined. Therefore, this study was undertaken to discern the association of fertility with SCAs in a child-bearing adult population.

With the advent of clinically validated single-nucleotide polymorphism (SNP)-based noninvasive prenatal testing that incorporates maternal and paternal genotypes [19], for the first time we can estimate SCA prevalence in over 140,000 fertile women and nearly 30,000 fertile men. To our knowledge, this is the largest reported estimation of X and Y chromosome aneuploidy in an international population, and is the first study to examine prevalence in fertile adults. Improved understanding of this incidence will facilitate prenatal counseling, diagnosis, and treatment, including the ability to address resulting fertility issues in individuals of child-bearing age.

## Methods

### Subjects and Sample Collection

This was a retrospective analysis of prospectively collected data. Consecutive blood samples were collected from 143,098 pregnant women undergoing routine prenatal screening using a SNP-based noninvasive prenatal screening test (Panorama, from Natera Inc., San Carlos, CA) from February 2013-July 2014. This study was deemed exempt by an institutional review board to perform data analysis on existing commercial data (Ethical & Independent Review Services Assigned Study ID: 14064–01). All subjects within this data set had been separately consented to receive Panorama testing for fetal risk only, and all identifiers were removed before analysis, thereby making additional consent unnecessary. Clinical indications for testing are presented in S1 Table and include advanced maternal age, abnormal/positive serum screening, potential hereditary disease affecting the fetus, suspected or known fetal abnormality, and poor prior pregnancy history (such as a prior aneuploid pregnancy). The algorithm is designed to determine fetal risk only for major aneuploidies and whole chromosome SCAs [20,21]. Accompanying paternal samples were collected for 29,848 cases. After removing the cases that either failed QC or were out of scope of the NIPT test (S3 Table), as well as non-paternity cases, 141,916 maternal and 29,336 paternal samples were analyzed for SCA determination.

### Sample Analysis

#### Standard NIPT Assay

Samples were processed and analyzed using validated molecular biology and bioinformatics methodology. All samples were analyzed for fetal copy number of chromosomes 13, 18, 21, X, and Y. Informed consent for the standard NIPT assay are limited to fetal risk of chromosome aneuploidy only (non-mosaic monosomy, trisomy, UPD). Individual maternal karyotypes are therefore not analyzed nor reported for the standard NIPT assay. As part of the standard assay, cell-free DNA was isolated from maternal plasma, maternal genomic DNA was isolated from the buffy coat, and paternal genomic DNA was isolated from a buccal swab sample [19]. Cell-free and genomic DNA was amplified using massively multiplexed polymerase chain reaction (PCR) targeting 19,488 SNPs. All samples were subjected to high-throughput sequencing (Illumina HiSeq 2500); sequencing results were analyzed using a proprietary algorithm which assesses the allelic patterns at specific and composite SNP sites of chromosomes 13, 18, 21, X and Y only [19–22]. The algorithm first predicts the expected distribution of the alleles at each SNP location for a plasma sample containing different fetal cell-free DNA fractions for each hypothesis (monosomy, disomy, or trisomy), using parental genotypes and crossover frequency data. Next, it compares the observed allele distributions to the expected allele distributions for each of the possible scenarios, relative likelihood of each scenario is computed, and scenario with the maximum likelihood is selected. Cell-free DNA samples were sequenced to a minimum depth of four million reads; genomic DNA samples were sequenced at 16% of the cell-free DNA depth-of-read. Cell-free DNA samples were required to pass previously described stringent quality control metrics [19].

#### Present Study

For the purposes of this study, the extant database was accessed in an anonymized fashion and analyzed to derive maternal and paternal chromosomal data. For X-chromosome SNPs, B-allelic frequency (BAF) was evaluated to determine if the copy number differed from the normal (defined to be disomy for maternal samples, monosomy for paternal samples). For example, the expected BAF for a heterozygous SNP is 50% for a disomy, whereas it is 33.3% or 66.6% for a trisomy. Moreover, the total amplification level is considered to determine the origin of the abnormality (a BAF of 33.3% could be a result of perfect trisomy as well as a mosaic deletion). Similar methods were used for Y-chromosome. Samples’ p and q arm determinations matched in all cases except for the two samples which were identified as aneuploid with the gain of two copies of the q arm of the X chromosome and normal diploid of the p arm consistent with 47,XX,+,i(X)(q10). Maternal samples with an identified sex chromosome aneuploidy did not generate a result for sex chromosomes in the case of a female fetus (no called), and parental genotypic results were not reported to patients. P-values were calculated using a Mann-Whitney rank sum test.

## Results

### Patient Demographics

Patient characteristic details for maternal and paternal samples are described in S2 Table; S1, S2, and S3 Figs depict maternal age, maternal weight, and gestational age distributions, respectively. Overall, mean maternal age was 33.4±5.8 years; 46.3% were ≥35 years of age. The population is a high-risk group due to advanced maternal age who were referred for NIPT. Mean gestational age at time of NIPT was 13.6±5.5 weeks. Gestational age of the pregnancy is not expected to affect maternal genotype. Mean maternal weight was 160.0±40.1 pounds. The majority of maternal and paternal samples originated within the United States (82.8% and 57.5%, respectively).

### Results and Population Incidence

Female X and Y chromosome results and population incidences are described in Table 1. Nearly all women (99.9%) in the cohort showed a 46,XX chromosome complement. SCA's prevalence in this cohort of women was 1 in 1,378. The most prevalent aneuploidy showed 45,X (including 45,X/46,XX mosaicism), occurring in 1 in 3,300 pregnant women, followed by 47,XXX (including 47,XXX/46,XX mosaics), occurring in 1 in 4,731 pregnant women. Note that using our methodology, it is not possible to differentiate between 45,X/46,XX and 45,X/47,XXX, therefore, 45,X/46,XX and 45,X/47,XXX incidences are combined. Twenty-three women (22.3% of maternal aneuploidies) were identified with X chromosome mosaicism of unknown type, including the clear presence of non-disomic cell lines but the ploidy state of all sub-types was not identifiable by our algorithm (i.e., the BAF was clearly different than a normal cell line but the total amplification levels were inconclusive), and could represent one or more different cell types. Three (2.9% of maternal aneuploidies) women were identified with 46,XX UPD (population prevalence: 1 in 47,305). As mentioned previously, two women (1.9% of maternal aneuploidies; population prevalence: 1 in 70 958) were identified with 4 copies of the q arm and 2 copies of the p arm, consistent with 47,XX,+i(X)(q10) chromosomal complement, a rare condition in which fertility is generally unimpaired [23,24]. Two women were identified with the presence of the Y chromosome, consistent with a 46,XY cell line, but the proportion of XY cells cannot be determined.

**Table 1:** Maternal and Paternal Genotypes and Population Incidences.

**Maternal Genotype (N = 141,916)**	**Total (N)**	**Observed Incidence, 1 in:**	**Mean (Median) Maternal Age**	**St. Dev. Maternal Age**	**p-value**^**1**^
46,XX	141,813		33.4 (34.6)	5.74	NA
45,X^2^	43	3,300	33.8 (33.5)	5.11	0.976
45,X	3	47,305	37.8 (34.2)	6.59	0.418
45,X/46,XX Mosaic^3^	40	3,548	33.5 (33.3)	5.23	0.800
47,XXX^4^	30	4,731	**30.9 (31.7)**	**5.71**	**0.023**
47,XXX	18	7,884	**30.0 (30.8)**	**5.96**	**0.016**
46,XX/47,XXX Mosaic	12	11,826	32.3 (33.9)	5.27	0.516
47,XX,i(X)(q10)	2	70,958	38.4 (38.4)	8.82	0.379
46,XX UPD	3	47,305	**23.4 (18.5)**	**9.66**	**0.048**
46,XY	2	70,958	35.3 (35.3)	0.29	0.732
Mosaic, unknown origin	23	6,170	35.1 (36.8)	6.88	0.079
**Total Aneuploid**	103	1,378	33.1 (33.5)	6.19	0.560
**Paternal Genotype (N = 29,336)**	**Total (N)**	**Observed Incidence, 1 in:**	**Mean (Median) Paternal Age**	**St. Dev. Paternal Age**	**p-value**
46,XY	29,320	-	-	-	-
47,XXY^5^	2	14,668	-	-	-
47,XXY	1	29,336	-	-	-
46,XY/47,XXY Mosaic	1	29,336	-	-	-
47,XYY^6^	10	2,934	-	-	-
47,XYY	9	3,260	-	-	-
46,XY/47,XYY Mosaic	1	29,336	-	-	-
Partial Y Deletion	4	7,334	-	-	-
**Total Aneuploid**	16	1,834	-	-	-

^1^p-value (one-tailed) calculated with respect to the 46,XX population for maternal aneuploidies

^2^Includes non-mosaic 45,X and mosaic 45,X/46,XX cases

^3^Of the 40 mosaic 45,X/46,XX cases, 22 (55.0%) had ≥50% monosomy.

^4^Includes non-mosaic 47,XXX and mosaic 46,XX/47,XXX cases

^5^Includes non-mosaic 47,XXY and mosaic 46,XY/47,XXY cases

^6^Includes non-mosaic 47,XYY and mosaic 46,XY/47,XYY case.

Maternal age was significantly associated with certain aneuploidies. 46,XX UPD women had a mean maternal age of 23.4 years, significantly lower than 46,XX women, who had a mean maternal age of 33.4 years (p = 0.048), however a sample size of two is not sufficient to make statistically significant conclusions. Similarly, 47,XXX women had a mean maternal age of 30.0 years, significantly lower than 46,XX women (p = 0.016). The ages of women with 45,X, 47,XXX, 48,XXXX and 46,XYchromosome complements, or with mosaicism of undetermined origin did not significantly differ from those women with a 46,XX genotype (p = 0.418, 0.800, 0.516, 0.379, 0.732 or 0.079, respectively). It is known that somatic mosaicism for the X chromosome aneuploidy in women increases with age, for example, for women above age 51, this rate is between 3.2 and 5.1% [25].

Male genotypes and population incidences are described in Table 1. Nearly all men (99.9%) were identified with a 46,XY genotype. Overall aneuploidy prevalence was 1 in 1,834. The most prevalent aneuploidy was 47,XYY or mosaic 46,XY/47,XYY, occurring in 1 in 2,934 males. Four men (1 in 7,334) were identified with a partial deletion on the Y chromosome; the chromosomal location could not be determined. Two men (1 in 14,668) were identified with a 47,XXY or mosaic 46,XY/47,XXY chromosomal complement.

### Noninvasive Prenatal Screening Results

None of the 103 women identified with an X chromosomal abnormality resulted in a fetal copy number call on the X chromosome, as the fetal copy-number algorithm is framed around maternal disomy. When there was an aneuploid maternal sample on the X chromosome, the algorithm is not able to compute a result for the fetal sample. One plasma sample from an affected mother showed a high-risk for fetal trisomy 21 (Table 2); the remaining cases with parental sex chromosome aneuploidy were low-risk for trisomy at chromosomes 13, 18 and 21. Five samples provided no results for the fetus on all tested chromosomes due to low fetal fraction (Table 2).

**Table 2:** Parental Aneuploidy & Corresponding Fetal NIPT Outcomes.

	Total (N)	Prevalence & Incidence				Corresponding NIPT^1^ Outcomes				
		Observed Incidence, 1 in:	Observed Prevalence per 100,000	Expected Prevalence per 100,000^2^	Fertility Estimate (Observed/Expected)	High-risk Result	Low-risk Result	No result, LFF^3^	No-result, Monosomy X	No-result, Unmatching Maternal Alleles
**Maternal Aneuploidy**										
**45,X or 45,X/46,XX**	43	3,300	30.3	29.3	103%	1	20	0	21	1
**47,XXX or 46,XX/47,XXX**	30	4,731	21.1	105.6	20%	0	14	3	13	0
**48,XXXX**	2	70,958	-	-	-	0	1	0	1	0
**Mosaic**^**4**^	23	6,170	-	-	-	0	12	2	8	1
**46,XX UPD**	3	47,305	-	-	-	0	1	0	2	0
**46,XY**	2	70,958	-	-	-	0	0	0	2	0
**Total**	103	1,378	-	-	-	1	48	5	47	2
**Paternal Aneuploidy**										
**47,XXY or 46,XY/47,XXY**	2	14,668	6.8	156.7	4%	0	2	0	0	-
**47,XYY or 46,XY/47,XYY**	10	2,934	34.1	117.5	29%	1	9	0	0	-
**Partial Y Deletion**	4	7,334	-	-	-	0	2	0	2	-
**Total**	16	1,834	-	-	-	1	13	0	2	-

^1^NIPT: Non-invasive Prenatal Testing

^2^Expected prevalence from Nielsen and Wohlert (1991); 34,910 newborns

^3^LFF: low fetal fraction

^4^Mosaics of unknown origin.

Of the 16 men identified with X or Y chromosomal aneuploidy, a high-risk fetal result (for 47,XYY) was determined in one case (Table 2). Remaining determinations were low-risk for all tested chromosomes, including at the X and Y chromosomes. No samples ended up with case level no-result calls due to low fetal fraction; however, two provided no results for Monosomy X because the paternal partial Y prevented fetal Y chromosome interpretation (Table 2).

### Fertility Estimates

**Fig 1:**
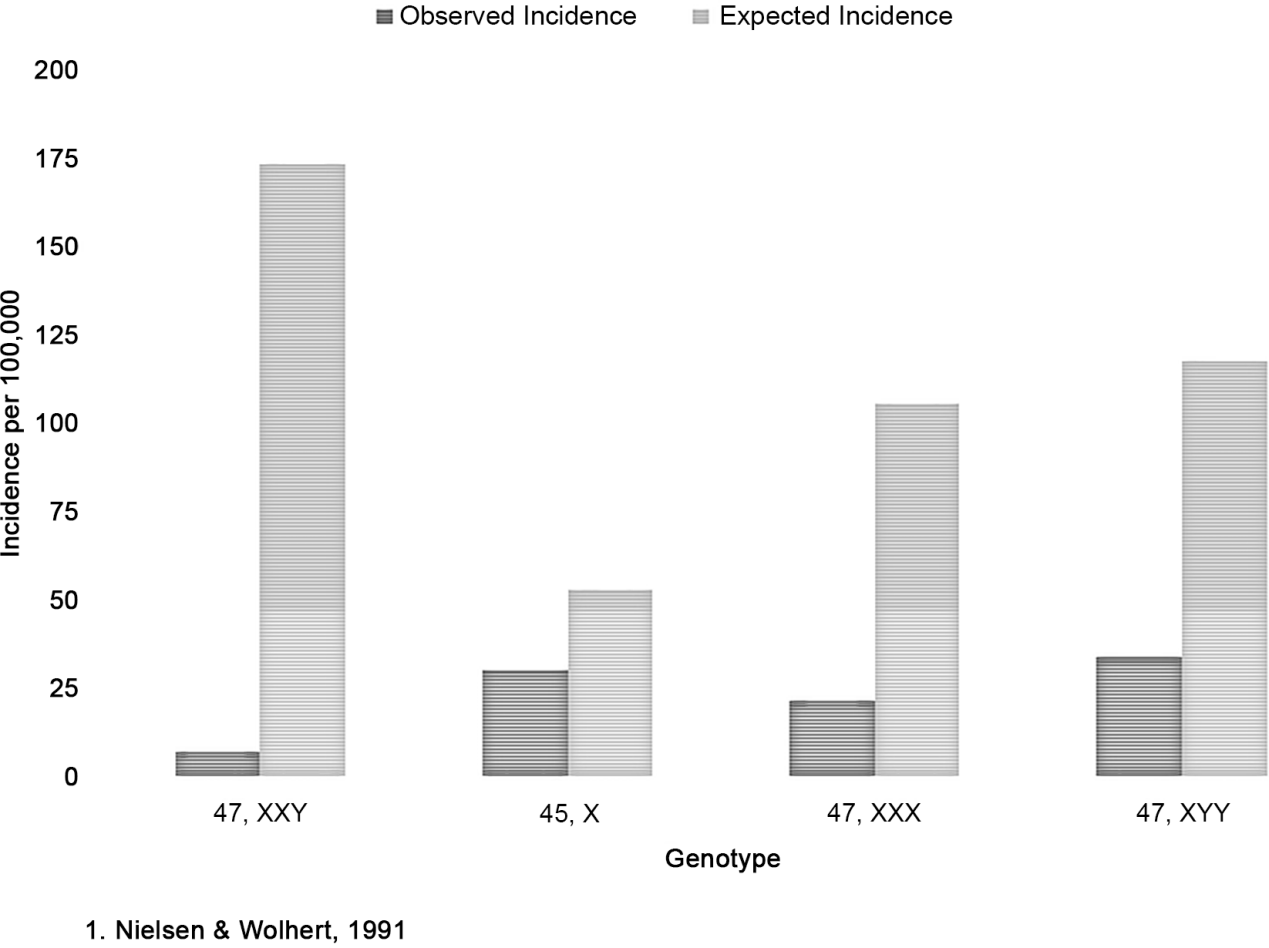
Incidence of X and Y chromosomal aneuploidy compared to previous studies.

X and Y chromosomal aneuploidy incidence in this cohort, as compared to previous incidence estimates, is depicted in Fig 1. Observed prevalence was less than expected for aneuploidies in this population. Assuming previous incidence estimates [1] to be accurate, fertility in this study would be expected in all females with mosaic 45,X, 20% of females with 47,XXX (and mosaics), 4% of males with 47,XXY (and mosaics), and 29% of males with 47,XYY (and mosaics). Additionally, 93% of 45,X mothers and 40% of 47,XXX mothers in this study were 46,XX mosaics, while 50% of the fertile men with 47,XXY and 10% of those with 47,XYY were 46,XY mosaics.

## Discussion

To our knowledge, this is the largest study to date estimating X and Y chromosomal aneuploidy prevalence in the child-bearing adult pregnant population using NIPT. In this large international population-based sample, 119 X and Y chromosomal abnormalities in maternal and paternal samples were identified, with an overall prevalence of 1 in 1,606 (adjusted to assume a 1:1 male:female ratio); this differs from early prenatal studies which estimated an incidence of 1 in 426 [1]. Assuming most of the individuals in this study are mosaic for one of the sex chromosome aneuploidies described, one would expect a large variability in the prevalence, depending on the degree of mosaicism present. For example, women with chromosomal complement of 45,X /46,XX were seen in 1 in 3,300, with a range of the abnormal cell line from 34% to >95%. Although women with 47,XXX are generally fertile, there is an increased risk of Premature Ovarian Failure (POF) in this disorder, in addition to reports of early ovarian or uterine dysgenesis [26,27]. In this study, we found an incidence of 1/4,731 which further supports POF and documents a distinct difference from prenatal incidences of 1/1000.

In this study, two cases of men with mosaicism for 47,XXY was observed, a prevalence of 1 in 14,668, and mosaicism for 47,XYY was 1 in 2,934. Natural fertility in men with 47,XXY is less common than in 46,XY men, as most 47,XXY men are azoospermic due to testicular failure in conjunction with hypogonadism [28]. Men with 47,XYY are also reported to have a four-fold increased infertility risk compared to men with 46,XY, although the etiology of this infertility remains unclear [29,30]. The findings here support the well-documented impact of an additional X-chromosome on male fertility and reproduction, and suggest that an extra Y chromosome has an impact, but less than an additional X [31].

Fertility in 47,XX,i,(X)(q10) is not well documented, though at least two successful pregnancies have been observed in this disorder [23]. 47,XX,i,(X)(q10) is not expected, necessarily, to lead to infertility, though secondary ovarian failure has been observed [24]. This is the first documentation of both incidence and fertility in this X chromosome structural rearrangement. Four cases of men with a partial Y deletion were seen, a prevalence of 1 in 7,334, which is the first study we are aware of to identify this abnormality in the fertile, child bearing population.

To our knowledge, there has been only one other study examining chromosomal anomalies in the fertile adult male population, and that study used gamete donor karyotypes [32]. In that study of 10,202 sperm donors, Ravel *et al*. documented five cases of 47,XYY (prevalence: 1 in 2,000) and two cases of 46,XY/47,XXY (>25% aneuploidy cells), corresponding to a prevalence of approximately 1 in 5,000 [32]; there were no documented fertile men with the non-mosaic 47,XXY genotype. This population-based sample of 29,336 fertile men identified nine as non-mosaic 47,XYY (prevalence: 1 in 3,260), and there was one case each of 46,XY/47,XYY, 46,XY/47,XXY, and non-mosaic 47,XXY (prevalence: 1 in 29,336). In the total male sample, we found that sex chromosomal aneuploidies occurred in 0.05% of the population, compared to 0.13% in Ravel *et al*., 2006. The lower percentage in this study may be partially explained by lower fertility among males with sex chromosome aneuploidies.

As this assay measures maternal and paternal genotypes using genomic DNA isolated from white blood cells, the possibility exists that fertility, and thus estimated prevalence, is confounded by isolated gonadal aneuploidy, and there is no information on how these genotypes are (or are not) correlated in mosaic individuals.

When considering factors that contribute to X and Y aneuploidy in the male cohort, studies have shown that sperm aneuploidy is associated with male infertility and poor semen quality, seen in approximately 2% of all men with fertility problems, 5% of oligozoospermic men, and 14% of azoospermic men [33–35]. This phenomenon leads to an increase in aneuploid spermatozoa which increases the risk of genetic abnormalities to embryos and offspring [36]. A recent study has shown that spermatozoa with aneuploidy of a sex chromosome can be longer-lived than spermatozoa with autosome aneuploidy [18]. Because long-lived aneuploid spermatozoa could have a greater chance of fertilizing oocytes, they may cause an increased risk of transmitting sex chromosome aneuploidies of paternal origin to their offspring [18,37].

When considering other factors that may have affected the frequency of X and Y aneuploidies observed in our fertile cohort, it is important to note that assisted pregnancy and conception techniques have become increasingly common in men and women with X and Y aneuploidies. It is estimated that up to 30% of 45,X women may achieve pregnancy using donor oocyte and in vitro fertilization [38]. Similarly, intra-cytoplasmic sperm injection (ICSI) and testicular sperm extraction (TESE) has led to over 100 reports of successful conceptions by men with 47,XXY [28]. We do not expect these techniques to have had a significant impact on the maternal results, as egg donor women were excluded. However, ICSI and TESE information were not collected on men submitting samples. Thus, we are unable to report whether the men found in this cohort with 47,XXY/46,XY who had a pregnant partner conceived spontaneously or artificially. Similarly, information regarding IVF or other fertility treatment was not collected for the women in this cohort.

As these samples were collected as part of routine NIPT, it is expected that both ascertainment and selection bias (excepting selection for the fertile population) were significantly minimized.

This study expands our understanding of the incidence of X and Y chromosomal aneuploidies in the adult fertile pregnant population and augments our knowledge of fertility in adults with these disorders, broadening our understanding of the fertility and prevalence of X and Y chromosomal aneuploidy.

## Supporting Information

S1 Fig Maternal age distribution. **A.** Distribution in the full cohort of samples. **B.** Distribution in samples with a 46,XX genotype. **C.** Distribution in samples with a non-mosaic 45,X or mosaic 45,X/46,XX genotype. **D.** Distribution in samples with a non-mosaic 47,XXX or mosaic 46,XX/47,XXX genotype. **E.** Distribution in samples with mosaicism of unknown origin.
(JPG)

S2 Fig Maternal weight distribution. **A.** Distribution in the full cohort of samples. **B.** Distribution in samples with a 46,XX genotype. **C.** Distribution in samples with a non-mosaic 45,X or mosaic 45,X/46,XX genotype. **D.** Distribution in samples with a non-mosaic 47,XXX or mosaic 46,XX/47,XXX genotype. **E.** Distribution in samples with mosaicism of unknown origin.
(JPG)

S3 Fig Gestational age distribution. **A.** Distribution in the full cohort of samples. **B.** Distribution in samples with a 46,XX genotype. **C.** Distribution in samples with a non-mosaic 45,X or mosaic 45,X/46,XX genotype. **D.** Distribution in samples with a non-mosaic 47,XXX or mosaic 46,XX/47,XXX genotype. **E.** Distribution in samples with mosaicism of unknown origin.
(JPG)

S1 Table Clinical indications for NIPT. (DOCX)

S2 Table Demographics of Study Population. (DOCX)

S3 Table Case Exclusion Criteria. (DOCX)
