# Supplementary figures and images for: Incidence of X and Y Chromosomal Aneuploidy in a Large Child Bearing Population
Source: PLoS One. 2016 Aug 11;11(8):e0161045. doi: 10.1371/journal.pone.0161045 (PMC4981345; doi:10.1371/journal.pone.0161045)

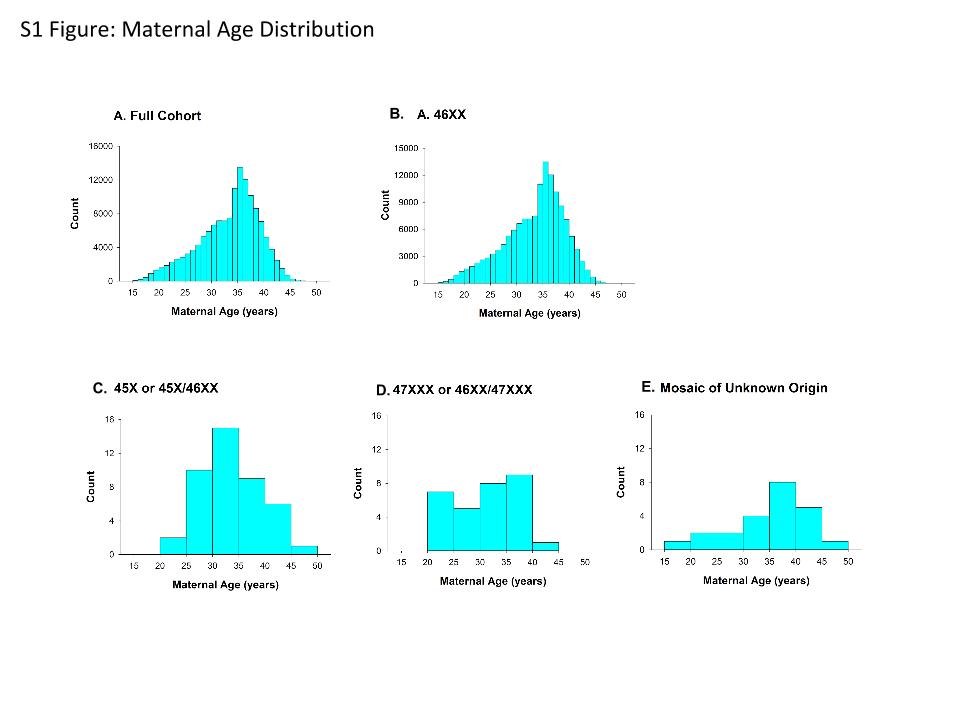

Supplement: S1 Fig — A. Distribution in the full cohort of samples. B. Distribution in samples with a 46,XX genotype. C. Distribution in samples with a non-mosaic 45,X or mosaic 45,X/46,XX genotype. D. Distribution in samples with a non-mosaic 47,XXX or mosaic 46,XX/47,XXX genotype. E. Distribution in samples with mosaicism of unknown origin. (JPG) [file pone.0161045.s001.jpg]

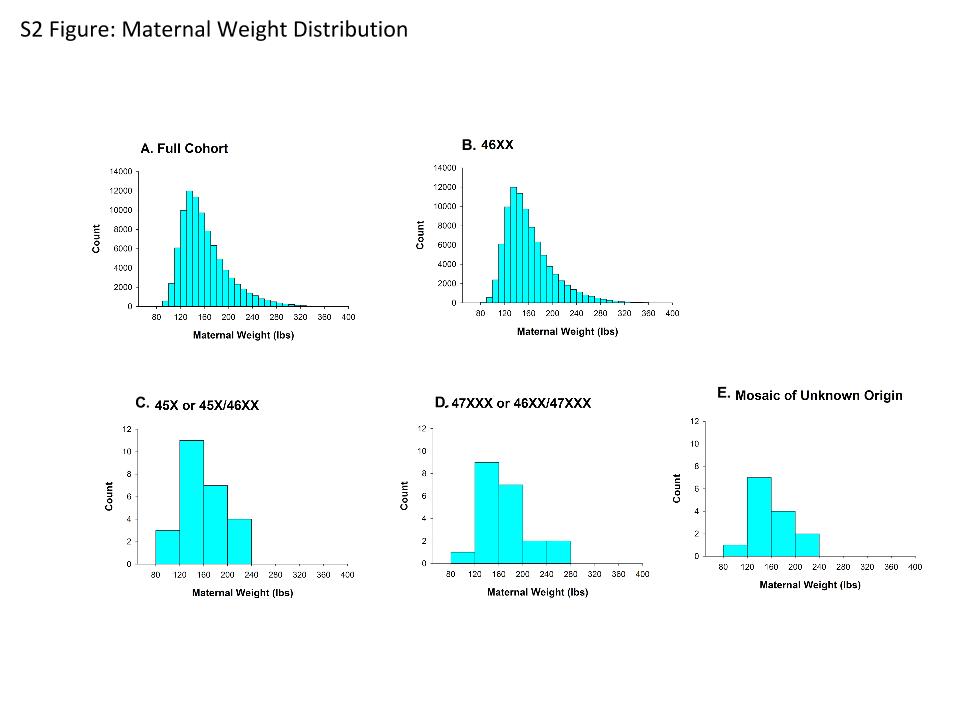

Supplement: S2 Fig — A. Distribution in the full cohort of samples. B. Distribution in samples with a 46,XX genotype. C. Distribution in samples with a non-mosaic 45,X or mosaic 45,X/46,XX genotype. D. Distribution in samples with a non-mosaic 47,XXX or mosaic 46,XX/47,XXX genotype. E. Distribution in samples with mosaicism of unknown origin. (JPG) [file pone.0161045.s002.jpg]

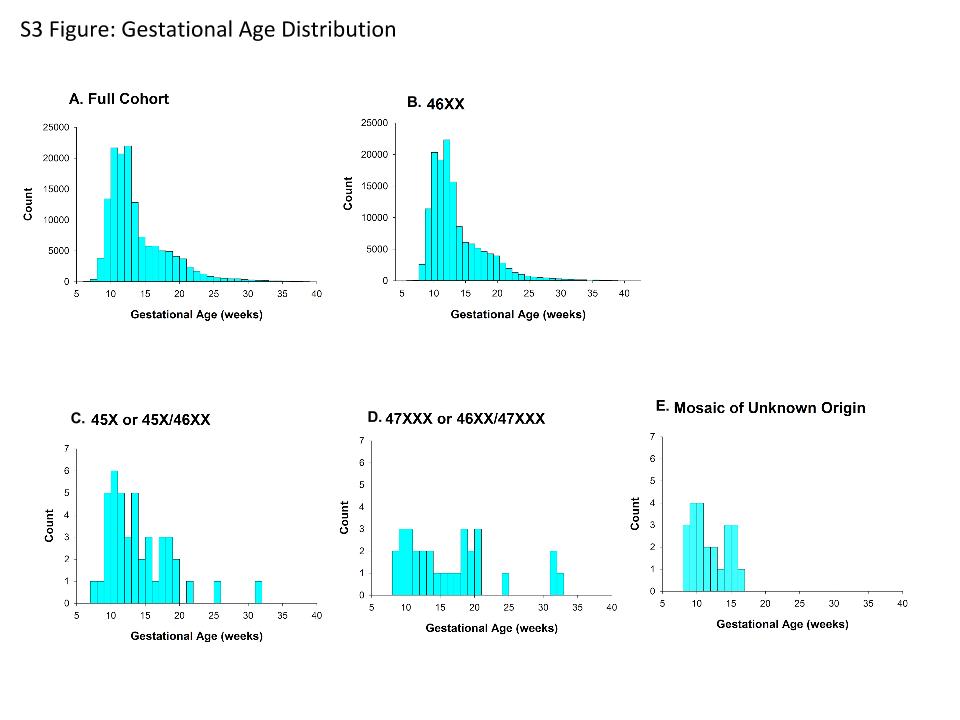

Supplement: S3 Fig — A. Distribution in the full cohort of samples. B. Distribution in samples with a 46,XX genotype. C. Distribution in samples with a non-mosaic 45,X or mosaic 45,X/46,XX genotype. D. Distribution in samples with a non-mosaic 47,XXX or mosaic 46,XX/47,XXX genotype. E. Distribution in samples with mosaicism of unknown origin. (JPG) [file pone.0161045.s003.jpg]
