# Supplementary material for: Incidence of X and Y Chromosomal Aneuploidy in a Large Child Bearing Population
Source: PLoS One. 2016 Aug 11;11(8):e0161045. doi: 10.1371/journal.pone.0161045 (PMC4981345; doi:10.1371/journal.pone.0161045)
Supplement: S1 Table — (DOCX) [file pone.0161045.s004.docx]

**S1 Table: Clinical indications for NIPT***

| **Possible Clinical Indications** |
| --- |
| Advanced maternal age (first pregnancy) |
| Advanced maternal age (not first pregnancy) |
| Abnormal/positive serum screening |
| Possible hereditary disease affecting fetus |
| Other specified antenatal screening |
| Other known or suspected abnormality in fetus affecting maternal management |
| Unspecified antenatal screening |
| Pregnancy with poor reproductive history (prior pregnancy with an aneuploidy) |
| Other (with request for relevant ICD-9 code) |

*NIPT: Non-Invasive Prenatal Testing
