# Supplementary material for: Incidence of X and Y Chromosomal Aneuploidy in a Large Child Bearing Population
Source: PLoS One. 2016 Aug 11;11(8):e0161045. doi: 10.1371/journal.pone.0161045 (PMC4981345; doi:10.1371/journal.pone.0161045)
Supplement: S2 Table — (DOCX) [file pone.0161045.s005.docx]

**S2 Table: Demographics of Study Population.**

|  | **Maternal**  **(N=141,916)** | **Paternal (N=29,336)** |
| --- | --- | --- |
| Age, years  Mean  Median  Range | 33.4±5.8  34.6  12.6-63.2 | NC^1^ |
| Country of Origin^2^ (n, %)  United States  Ex-United States^3^ | 117,513 (82.8%)  24,363 (17.2%) | 16,856 (57.5%)  12,480 (42.5%) |
| Gestational Age (wks)  Mean  Median  Range | 13.6±5.5  12.1  5.7-40.9 | NA |
| Weight (pounds)^4^  Mean  Median  Range | 160.0±40  151.0  80.0-460.0 | NA |

^1^Information not collected.

^2^Top five most prevalent countries of origin

^3^No country reported for 418 maternal and 149 paternal samples.

^4^Weight reported only for the cases originating from within the United States to avoid including weights reported in Kg
