# Supplementary material for: Incidence of X and Y Chromosomal Aneuploidy in a Large Child Bearing Population
Source: PLoS One. 2016 Aug 11;11(8):e0161045. doi: 10.1371/journal.pone.0161045 (PMC4981345; doi:10.1371/journal.pone.0161045)
Supplement: S3 Table — (DOCX) [file pone.0161045.s006.docx]

**S3 Table: Case Exclusion Criteria**

| **Exclusion Criteria** | **Number Excluded** |
| --- | --- |
| *Quality Control* |  |
| Insufficient blood volume (<13 mL) | 276 |
| Incorrect collection tube | 19 |
| Sample damage | 12 |
| Missing Patient Information | 149 |
| Known multiple gestation | 75 |
| Sample received more than 6 days after collection | 253 |
| Confirmed donor egg pregnancy | 39 |
| Surrogate carrier | 4 |
| Low gestational age | 324 |
| Other | 31 |
|  | **Total: 1,182** |
